# Cognition, culture and utility: plant classification by Paraguayan immigrant farmers in Misiones, Argentina

**DOI:** 10.1186/s13002-017-0169-4

**Published:** 2017-07-25

**Authors:** Monika Kujawska, N. David Jiménez-Escobar, Justin M. Nolan, Daniel Arias-Mutis

**Affiliations:** 1 Institute of Ethnology and Cultural Anthropology, Lodz University, Lindleya 3/5, 90-131, Lodz, Poland; 2 CONICET- IDACOR, Museo de Antropología, Universidad Nacional de Córdoba, Av. Hipólito Yrigoyen 174, Córdoba, Argentina; 3 Department of Anthropology, Old Main 330, University of Arkansas, Fayetteville, AR 72701 USA; 4 Artificial Intelligence, Rijksuniversiteit Groningen, Netherlands, Carrera 50 Bis # 44A-32, Bogotá, Colombia

**Keywords:** Rural communities, Medicinal plants, Wild edible plants, Folk epistemology

## Abstract

**Background:**

This study was conducted in three rural communities of small farmers of Paraguayan origin living in the province of Misiones, Argentina. These *Criollos* (Mestizos) hail chiefly from departments located in the east of Paraguay, where the climate and flora have similar characteristics as those in Misiones. These ecological features contribute to the continuation and maintenance of knowledge and practices related to the use of plants.

**Methods:**

Fieldwork was conducted between September 2014 and August 2015. Forty five informants from three rural localities situated along the Parana River participated in an ethno-classification task. For the classification event, photographs of 30 medicinal and edible plants were chosen, specifically those yielding the highest frequency of mention among the members of that community (based on data obtained in the first stage of research in 2014). Variation in local plant classifications was examined and compared using principal component analysis and cluster analysis.

**Results:**

We found that people classify plants according to application or use (primarily medicinal, to a lesser extent as edible). Morphology is rarely taken into account, even for very similar and closely-related species such as varieties of palms. In light of our findings, we highlight a dominant functionality model at work in the process of plant cognition and classification among farmers of Paraguayan origin. Salient cultural beliefs and practices associated with rural Paraguayan plant-based medicine are described. Additionally, the manner by which residents’ concepts of plants articulate with local folk epistemology is discussed.

**Conclusions:**

Culturally constructed use patterns ultimately override morphological variables in rural Paraguayans’ ethnobotanical classification.

## Resumen

**Antecedentes:**

Este trabajo se realizó con pequeños agricultores de origen paraguayo, que habitan en la provincia de Misiones, Argentina. Los *criollos* (mestizos) en su mayoría provienen de departamentos ubicados al oriente del Paraguay, donde el clima y la flora presentan características similares a la provincia de Misiones. Estas características ecológicas contribuyen a la continuación y el mantenimiento de los conocimientos y las prácticas relacionados al uso de las plantas.

**Métodos:**

El trabajo de campo se realizó entre septiembre de 2014 y agosto de 2015. En la etnoclasificación participaron 45 informantes, provenientes de tres localidades rurales, situadas a lo largo del río Paraná, frontera entre los dos países. Para la clasificación se utilizaron imágenes fotográficas de 30 especies -comestibles y medicinales- preseleccionadas como las de mayor frecuencia de mención entre los habitantes de la región (a partir de los datos obtenidos en una primera etapa de investigación en el año 2014). Por medio del análisis de componentes principales y el análisis de agrupamiento (cluster) se contrastaron y compararon las variaciones en las etnoclasificaciones locales de plantas.

**Resultados:**

Se encontró que los pobladores clasifican sus plantas según su aplicación y uso (mayormente asociadas a la categoría medicinal y en menor medida a la comestible). Mientras, la morfología rara vez se tiene en cuenta, incluso en aquellas especies muy similares y estrechamente relacionadas como las palmeras. A la luz de los hallazgos, se destaca un modelo de funcionalidad dominante en el proceso de cognición y de clasificación de las plantas entre los agricultores de origen paraguayo. Se describen las principales creencias y prácticas culturales asociadas a la medicina rural paraguaya. Adicionalmente, se discuten algunos de los conceptos de las plantas que mantienen los paraguayos y su articulación con la epistemología local.

**Conclusiones:**

En la clasificación etnobotánica de los pobladores de origen paraguayo los patrones de uso construidos culturalmente tienen mayor peso que las variables morfológicas.

## Background

For decades, scholars have attempted to understand the principles underlying folk biological classification, including different ways of organizing categories of living things in the natural world [1]. Does classification serve cognitive or utilitarian ends, a mix of both, or neither [2]? The debate bridges two influential schools of thought in academic anthropology: Malinowski’s functionalism and Lévi-Strauss’ structuralism [3]. The former claimed, in short, that knowledge of indigenous people had adaptive significance and was oriented towards human survivorship. Lévi-Strauss proposed a contrasting reason by which indigenous people classify nature for cognitive, or intellectual purposes, which were not seen as purely utilitarian but guided in essence by abstract thinking, a distinctly and uniquely human attribute [4].

From the 1970s through the 1990s, work in ethnotaxonomy by anthropologists and ethnobiologists shed powerful new light on how human beings understand and interact with plant and animal worlds. Berlin [5, 6] and Brown [7, 8] advanced the implicitly intellectualist viewpoint that human minds are inclined to recognize ‘natural discontinuities’ in plant and animal domains, whereby ambient flora and fauna are recognized, named, and classified according to easily perceptible morphological cues. Indigenous nomenclature and classification systems have also been interpreted as largely functional and utilitarian in construction and design, however, predicated on the culturally-constructed “use value” attached to living things [9, 10] and their associated “activity contexts” [11].

The prevalence of abstract thought in human cognition and information processing was further extended by Ingold [12] who examined the division between hikers and tourists, and their level of interest in exploration of local environments, and is maintained presently by some contemporary researchers in ethnobiology [13]. The utilitarian approach was nourished by consensus analysis, in which knowledge was measured as a function of agreement between informants and variation in knowledge was not taken into account [14]. Newer studies put more attention on variation of knowledge in the study communities. However, the conclusions were that variation in folk category construction and in folk classification could be explained by the level of expertise of the classifier [15–18]. Nolan discovered that experts (herbalists, medical practitioners, conservation activists, shopkeepers who marketed botanical paraphernalia) relied on a combination of functional (utilitarian) and morphological cues when classifying wild plants, while novices were guided primarily by morphological cues. However, as a group they shared a cohesive system of classification of these resources. Other authors showed that familiarity, cultural competence, experience and interest play important roles in category construction and in folk classification [19]. Therefore, folk plant classification is believed in some cases to be organized around perceived characteristics, which are *both* perceptual and functional attributes that characterize plant species [17]. Soares et al. [20] point that the classification of living beings with consideration to their biological characteristics, such as morphology and life-form has been observed in some studies developed in Brazil. The preponderance of perceptual (morphological) characteristics in plant classification was explained as strong influence by Berlinean ideas, as seen in the works of Mourão and Montenegro [21] and Souza and Begossi [22].

How do human goals, activities and knowledge effect their classification strategies? Goal-derived categories are based on utilitarian concerns. Goals (needs) serve to activate context-dependent properties [11]. Extensive use of goal-derived categories by a given society or study group is revealed during sorting activity by linking justifications for groupings to goals. In this case the folk-taxonomic structure may resemble a series of relevant goal categories [23]. However, as Medin and colleagues suggested [19], goals do not necessarily partition the full set of entities in a domain or an entire classification, hence folk-classification may reflect a mixture of goal-derived and perceptual/morphological categories.

In our contribution we do not attempt to solve the problem of the supremacy of cognitive purposes over utilitarian ones (or vice versa) in category construction and folk classification. Rather we point to the complexity of the problem. The design of our research was not oriented towards general purpose classification [1], but rather to special purpose classification, which orients the results towards goal-derived classification [19, 24]. We chose 30 most salient species used as food and medicine by Paraguayan migrants living in the north-eastern Argentina. These plants could be classified as core species for the study community [24]. Therefore, our aim was to determine whether study participants could reveal strategies or approaches other than utilitarian categorisation, which would go beyond simple differentiation between medicinal and food plants. Moreover, we were interested how pile sorting task may contribute to the triangulation of information obtained previously during free elicitation/free listing procedure [25]. In addition, we aimed to ascertain whether pile sorting might reveal some semantic dimensions of relevance to the study community which were covert during the standard structure interviews. The specific goals, therefore, were to answer the following questions: 1) what features are considered most salient in plant classification? 2) how are categories of plants constructed culturally? 3) how does gender influence the plant classification, if at all? We hypothesize that there is one dominant pattern of plant classification which relies on useful features of plant resources, and which does not depend on exclusively morphological cues.

## Methods

### Study sites

Since the 19^th^ century, *Criollos* have been coming to Misiones from the south of Argentina (Corrientes) and from neighboring Brazil and Paraguay. Analysis of census data shows that since 1947, Paraguayans have comprised the most numerous migrant community in Argentina among neighbouring countries, and since 2001 they have been the most populous migrant community in Argentina, in general terms. According to the census from 2010, the Paraguayan community in Argentina accounts for 550.713 persons [26]. This figure represents 30.5% of all immigrants in the country, 1.4% of Argentina’s population and it accounts for approximately 8.6% of the total population of Paraguay [26, 27]. Until the mid-20^th^ century it was basically rural, unskilled migrants arriving to work on cotton or yerba mate plantations. Since the mid-20^th^ century, big cities started attracting Paraguayan migrants [27]. The push and pull factors for migration were both economic and political in character. There have been two major reasons for migration: the consequences of the Chaco War (1936) and the Paraguayan Civil War occurred in 1947. Decades of military dictatorship in Paraguay led to the expulsion of the population, and at the same time attracted people seeking agricultural employment opportunities in the production of cotton, sugar cane, tea and tobacco in the Argentine northeast. Meanwhile, the second flow occurred during and after the mid-1950s and was fueled by economic reasons; migrants moved to the metropolitan capital city, Buenos Aires [28].

Paraguayan migrants have been regarded as having low formal educational levels, especially those migrating to Argentina in the 1970s, who comprise the majority of our study population. In 1970, census data showed that half of the Paraguayan population residing in Argentina had less than 4 years of formal education. Since the 1990s males have moved into small-scale construction and farm labor, while women found work in domestic service [27]. It can be added from our field observations that in Misiones, Paraguayan men have found employment predominantly in the forest: in clearing the land, felling of trees and by working in forest plantations of pine and eucalyptus.

Given that in Misiones the natural environment is generally better preserved than it is in Paraguay [29, 30], Paraguayan migrants originating from the same ecoregion of the Atlantic Forest presumably have found better conditions for implementing and transmitting their traditional ecological knowledge than they did in their own country of origin.

### Data gathering

**Fig. 1:**
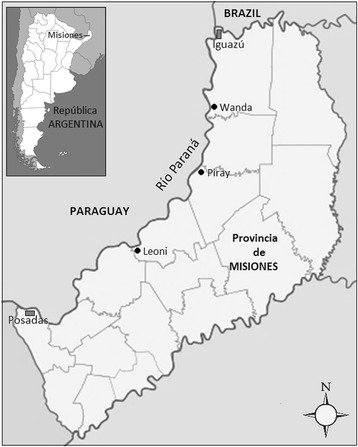
Distribution of the study localities, Misiones, Argentina

The research was conducted twofold. In the first phase, between September and November 2014, we worked with 71 informants (45 women aged between 30 – 95 and 26 men aged between 45 – 90) and conducted free-list interviews [31]. We asked them to list the edible and medicinal plants they knew, and the corresponding uses for each. Based on these preliminary results to frequency calculations, the 30 most cited species were chosen (Table 1). Some of the species were mentioned in both domains (edible and medicinal); in this case the number of citations was merged for a given species. In the second stage, we prepared 30 plant photos containing all key characteristics (fruits, flowers, foliage and bark) that local people might use to recognize them. These photos were taken in 2014 during the first stay in the field. The photos were labelled with local folk names in Spanish and Guarani languages. All plants were identified to the species level and corresponding “folk generic” level [19]. Altogether 45 persons (25 women and 20 men) volunteered to participate in the pile sorting task [32]: 13 in Puerto Leoni (hereinafter Leoni), 16 in Piray Km 18 (hereinafter Piray), and 16 in Puerto Wanda and Wanda town (hereinafter Wanda) (Fig. 1). Forty five participants in the pile sorting task belonged to the same pool of informants (71) who had taken part in the free listing task in the first stage of fieldwork. We did not choose the most knowledgeable or expert people from each community. All the study participants were asked for oral prior informed consent. There was no requirement for the project to pass any ethical commission, either in Argentinean or Polish institutions.

**Table 1:** Medicinal and edible plant species used in the pile-sort task among Paraguayan community, Misiones, Argentina

Common name	Scientific name	Botanical family name	Use
achicoria	*Hypochaeris chillensis* (Kunth) Hieron.	Asteraceae	food, medicinal
ajenjo	*Artemisia absinthium* L.	Asteraceae	medicinal
aloe	*Aloe maculata* All.	Asphodelaceae	medicinal
ambay	*Cecropia pachystachya* Trécul	Urticaceae	medicinal
araticú	*Rollinia salicifolia* Schltdl.	Annonaceae	food
cangorosa	*Maytenus ilicifolia* Mart. ex Reissek	Celastraceae	medicinal
carqueja	*Baccharis trimera* (Less.) DC.	Asteraceae	medicinal
cedrón	*Cymbopogon citratus* (DC.) Stapf.	Poaceae	medicinal
coco	*Acrocomia aculeata* (Jacq.) Lodd.	Arecaceae	food, medicinal
cocú	*Allophylus edulis* (A. St.-Hil., A. Juss. & Cambess.) Hieron. ex Niederl.	Sapindaceae	food, medicinal
guayaba	*Psidium* spp.	Myrtaceae	food, medicinal
guavirá	*Campomanesia xanthocarpa* Mart. ex O. Berg	Myrtaceae	food, medicinal
guembé	*Philodendron bipinnatifidum* Schott	Araceae	magical, medicinal, ornamental
ingá	*Inga marginata* Willd.	Fabaceae	food
isipó milhombres	*Aristolochia triangularis* Cham	Aristolochiaceae	medicinal
jacaratiá	*Jacaratia spinosa* (Aubl.) A. DC	Caricaceae	food
ka’a re	*Dysphania ambrosioides* (L.) Mosyakin & Clemants	Chenopodiaceae	medicinal
malva blanca	*Sida cordifolia* L.	Malvaceae	medicinal
manzanilla	*Matricaria chamomilla* L.	Asteraceae	medicinal
marcela	*Achyrocline tomentosa* Rusby	Asteraceae	medicinal
menta	*Mentha spicata* L.	Lamiaceae	medicinal
naranja	*Citrus sinensis* (L.) Osbeck	Rutaceae	food, medicinal
pacurí	*Rheedia brasiliensis* (Mart.) Planch. & Triana	Clusiaceae	food
pindó	*Syagrus romanzoffiana* (Cham.) Glassman	Arecaceae	food, medicinal
pitanga	*Eugenia uniflora* L.	Myrtaceae	food, medicinal
romero	*Rosmarinus officinalis* L.	Lamiaceae	food, medicinal
ruda	*Ruta chalepensis* L.	Rutaceae	magical, medicinal
salvia	*Lippia alba* (Mill.) N.E. Br.	Verbenaceae	medicinal
verbena	*Verbena montevidensis* Spreng.	Verbenaceae	medicinal
yva hai	*Eugenia pyriformis* Cambess.	Myrtaceae	food, medicinal

**Fig. 2:**
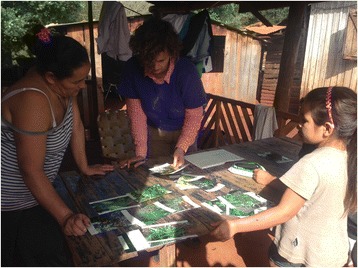
Documentation of pile sorting at Puerto Leoni, Misiones, Argentina, 2015

**Fig. 3:**
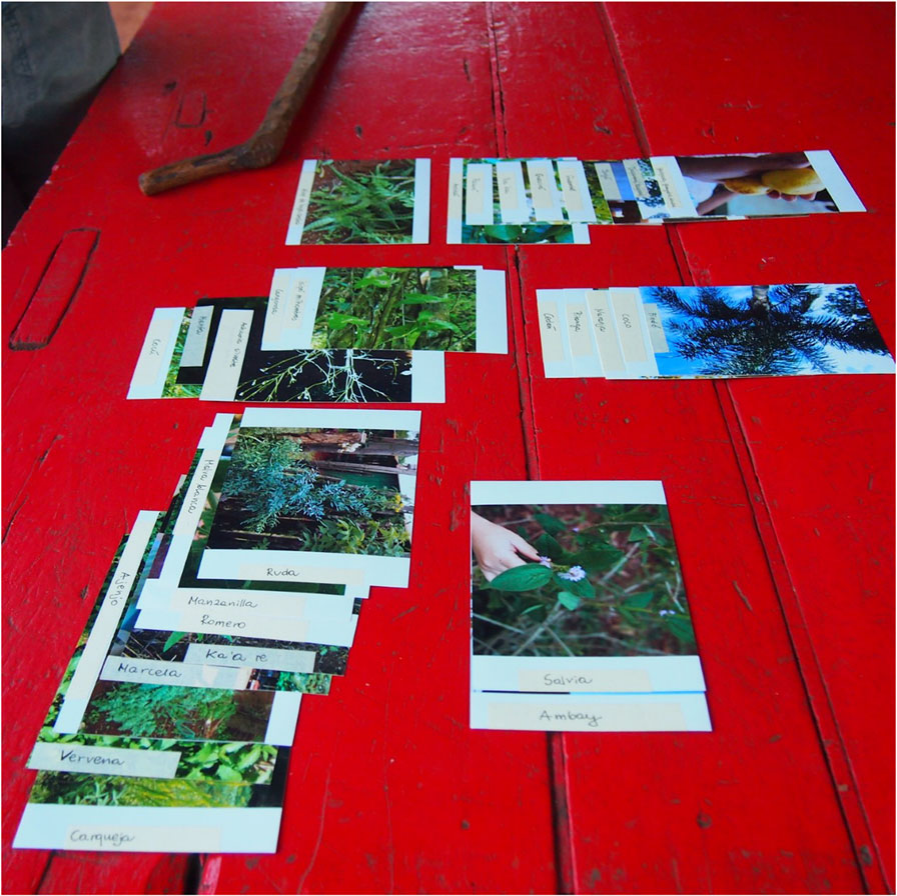
Pile sorting, Piray Km 18, Misiones, Argentina, 2015

Lay terminology was used to explain the aim and purpose of the pile sorting to the interlocutors. The discourse was developed around the notion of plants that “go together” for any reason deemed meaningful; plants considered similar to each other, based on any condition or feature, or plants that are seen to be kin. We endeavoured to provide the same explanations for all the participants. Each time a participant arranged a pile, she/he was asked for a justification of sorting plants together (Fig. 2). The information was written down into the field notes and a photo documentation was also made (Fig. 3).

### Data analysis

In this analysis we used data from the initial pile sorting called here time A.

#### Clustering

The cluster analysis was performed using Python-based SciPy Library of software [33]. In order to establish the relationship between 30 selected plants and the groups formed by each participant, an absence-presence *M*_*ij*_ matrix was constructed which included *i* rows and *j* columns. Each *i* row corresponds to a given informant’s piles, while the *j* column corresponds to a given species; if *j* plant is in the group of a given informant, it acquires (1) for a presence, and (0) for its absence.

By using the *M*_*ij*_ matrix the cluster analysis was conducted by applying the multidimensional Minkowski distance. This allowed the matrix of similarity between each of the 30 plant species to be constructed. Cluster analysis was then performed by using as the proxy the minimum distance between two branches.

#### Principal component analysis and K-means with covariance matrix

For the construction of the covariance matrix, the plant species vectors were studied (columns of matrix *Mij*), and the following rule was used: *Covij=E [(Xi- μi) (Xj- μj)* ^*T*^ *]*, in which *E* corresponds to the expected value; *X*_*i*_ and *X*_*j*_, are plant species vectors; μ_i_ and μ_j_ are the expected value of the row columns in matrix *Mij* (the expected value of a given informant group).

Principal component analysis (PCA) was performed upon the covariance matrix.. It consists, in short, of establishing the eigenvalues and eigenvectors of the *Cov*_*ij*_ matrix. Once calculated, the eigenvectors with the highest eigenvalues were selected and used to project the plants’ vectors. In this particular case, the first two eigenvectors (the first two components), covering more than 70% of the variability of the sample (*y*_1_=65.21%, *y*_2_=8.29%) were chosen.

The calculation of the values in the selected eigenvectors was followed by the grouping method using the *K-means*. The *K-means* algorithm separates the sample into *k* groups. The algorithm establishes a number of *k* centroids, measures the distance from the centroids to each plant, assigns each plant to the closest centroid group, and the position of the centroids are re-calculated at the center of each group. The algorithm iterates seeking optimal classification, which minimizes the cumulative distance of the sample to the centroids.

In order to set the optimal *k* number of groups, the changing accumulated distance towards centroid was explored with values between 2 and 10. The optimal value is set to the point after which the cumulative distance to centroid is stable [34], in this case *k* = 8. Also, a histogram of the number of piles arranged by each participant was elaborated. Based on this information, principal component analysis (PCA) was performed using the covariance matrix. It shows a classification of 30 plant species using the K-means algorithm with *k* = 8.

#### Content analysis

Content analysis [35, 36] conveys justifications provided by informants every time they arranged a pile (grouping) during the pile sorting task. All the reported justifications were entered into a database to quantify them (the same procedure as in a case of frequency of mention), and by doing so, to identify the most frequent explanations for grouping plants together.

## Results

### Salient features considered in plant classification

#### Grouping plants: community level

**Fig. 4:**
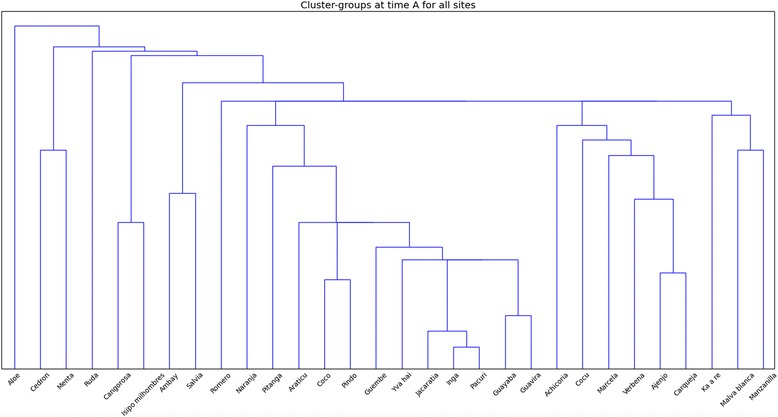
Cluster-groups at time A for all sites

Figure 4 presents the histogram for the three study localities altogether. Fruit plants form the most proximate group. Plants such as *ingá*, *pacurí*, *jacaratiá* (right of the center) exhibit the greatest proximity. Among the study population there is a great consensus to perceive these ethnotaxa as wild, forest fruit trees. Other closely clustered pairs form *guavirá* and *guayaba* –both used as fruit snacks primarily, and secondarily for the leaves’ astringent properties. They both belong to the Myrtaceae family and are also perceived as similar to each other. However, other species from the same botanical family, *yva hai* and *pitanga* (center) are loosely connected, especially *pitanga*, which exists on the cusp of medicinal and edible plants. Two palms, *coco* and *pindó* (center right), are clustered together. Although these species are used as both food and medicine, here they have been associated with edible species and their proximity may also be the result of their perceived morphological similarity. Starting from *achicoria*, on the right, there are two subgroups of medicinal species, all of which are used for the digestive system: *achicoria*, *cocú*, *marcela*, *verbena*, *ajenjo*, *carqueja*, *ka’a re*, *malva blanca*, *manzanilla*. The first two species in this group, *achicoria* and *cocú*, represent the gradient from edibility to medicinal in use-patterns; however, these species are considered more medicinal than edible. *Ajenjo*, *carqueja* and *verbena* reveal the greatest proximity in this group. They are considered the most bitter of all, and are clustered together due to this sensorial perception shared by many of the study participants. At the left side of the fruit trees cluster medicinal plants are grouped; however the first two of them, *pitanga* and orange (*naranja*), are also used for their edible fruits. In this group there are three easily visible clusters: *cangorosa* and *isipó milhombres* used as blood cleansers, *salvia* and *ambay* very potent cough expectorants, mint (*menta*) and lemon grass (*cedrón*) which are grouped together for different reasons such as not being bitter, having relaxing properties, and perceived as good additives for mate drinks. Aloe (*aloe*) (far left) stays on its own, as it was rarely grouped with other species. It was explained to us that aloe was a medicine which had to be used topically and could not be digested, therefore it could not be used as medicinal, edible, or an appropriate additive to mate and *tereré* (hot and cold infusion of *Ilex paraguaryensis*) in a way that most of the other sorted plant species could be used.

#### Grouping plants on a locality level

**Fig. 5:**
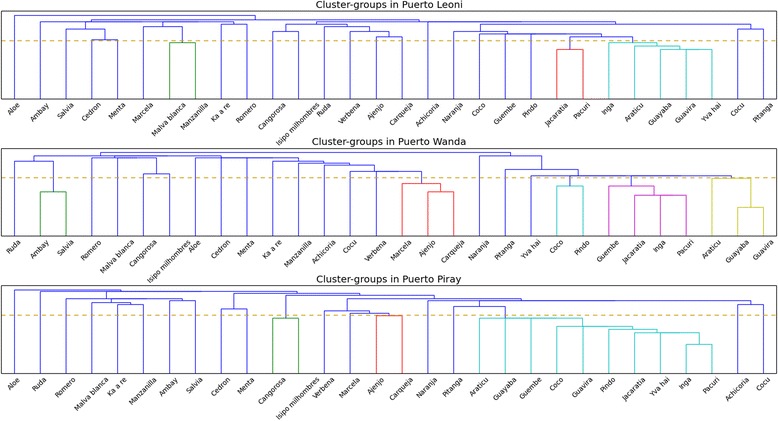
Cluster-groups in Puerto Leoni, Puerto Wanda, and Puerto Piray

In the second step of analysis we aimed to check whether locality differences existed in plant classification between the study sites. Figure 5 presents three separate cluster histograms for the study localities. In all three places we observe a similar pattern, namely that all plants were clustered according to their perceived functionality. In all three histograms wild edible plants are grouped on the right hand side, and medicinal ones on the left. In Leoni a species “mediating” between two groups is *achicoria* (center-right)*—*perceived as both edible and medicinal. In Wanda and Piray, orange *(naranja*), plays this role. In Leoni and Piray, aloe (far left) appears on its own. In all three places we observe that forest fruit bearing trees go together: in Leoni these are *jacaratiá* and *pacurí*, in Wanda *pacurí*, *ingá* and *jacaratiá* and in Piray *ingá* and *pacurí*. Some Myrtaceae species also were clustered in a similar way as in the general cluster: *guayaba* and *guavirá* in Wanda, *guayaba*, *guavirá* and *yva hai* in Leoni. As for medicinal plants, *cangorosa* an *isipó milhombres* were always clustered due to their well-defined properties as blood cleansers. Bitter plants such as *verbena*, *ajenjo*, *carqueja* and *achicoria* were grouped together. “Hot” remedies were clustered too, especially *malva blanca* and *manzanilla*, but this might also be due to their function as women’s plants, suitable for postpartum treatment and also during menstruation. Lemon grass (*cedrón*) and mint (*menta*) were grouped in all places the same as they were in the general cluster. Only in Wanda were two palms clustered—*coco* and *pindó* (right of the center). All in all, while some local particularities were found in each place, several similar patterns can be observed overall in plant classification across the study sites. They follow the overall structure observed previously in the general cluster.

On one occasion, an informant understood the task in such a way that she prepared several medical prescriptions based on mixing different herbs (by grouping together several plants). She followed the idea of balancing their “hot” and “cold” properties, hence grouping together plants with opposite properties. It should be explained here that the informant had left Paraguay 2 years before and she was still in the process of creating her medicinal home garden and rediscovering familiar medicinal plants in the surrounding. Therefore, she seemed very pleased by the prospects of freeing her imagination and creativity by preparing plant mixtures she desired in a real life but was constrained by the lack of proper resources. This example is also interesting because it shows that a grouping of similar plants may be formed by species with ostensibly opposite qualities; nonetheless these species may form groups based on their functional complementarity. The same is true for pairs: on a few occasions informants stated that plants in general have partners/live in pairs: male and female (*achicoria* male and female, rue (*ruda*) male and female, papaya (*mamón*) male and female, etc.). They also form groups, however composed of opposite “sexes”. The differences occur within groups and between groups. Hence, further research is needed to state what a nucleon (core) is and where a boundary of a group, kin or “a family” is. It can be added on the margin that in perceiving sexes among folk taxa, the study group mixes folk beliefs with biologically grounded observations, which are in agreement with biological science.

#### Revealing new groupings through principal component analysis

**Fig. 6:**
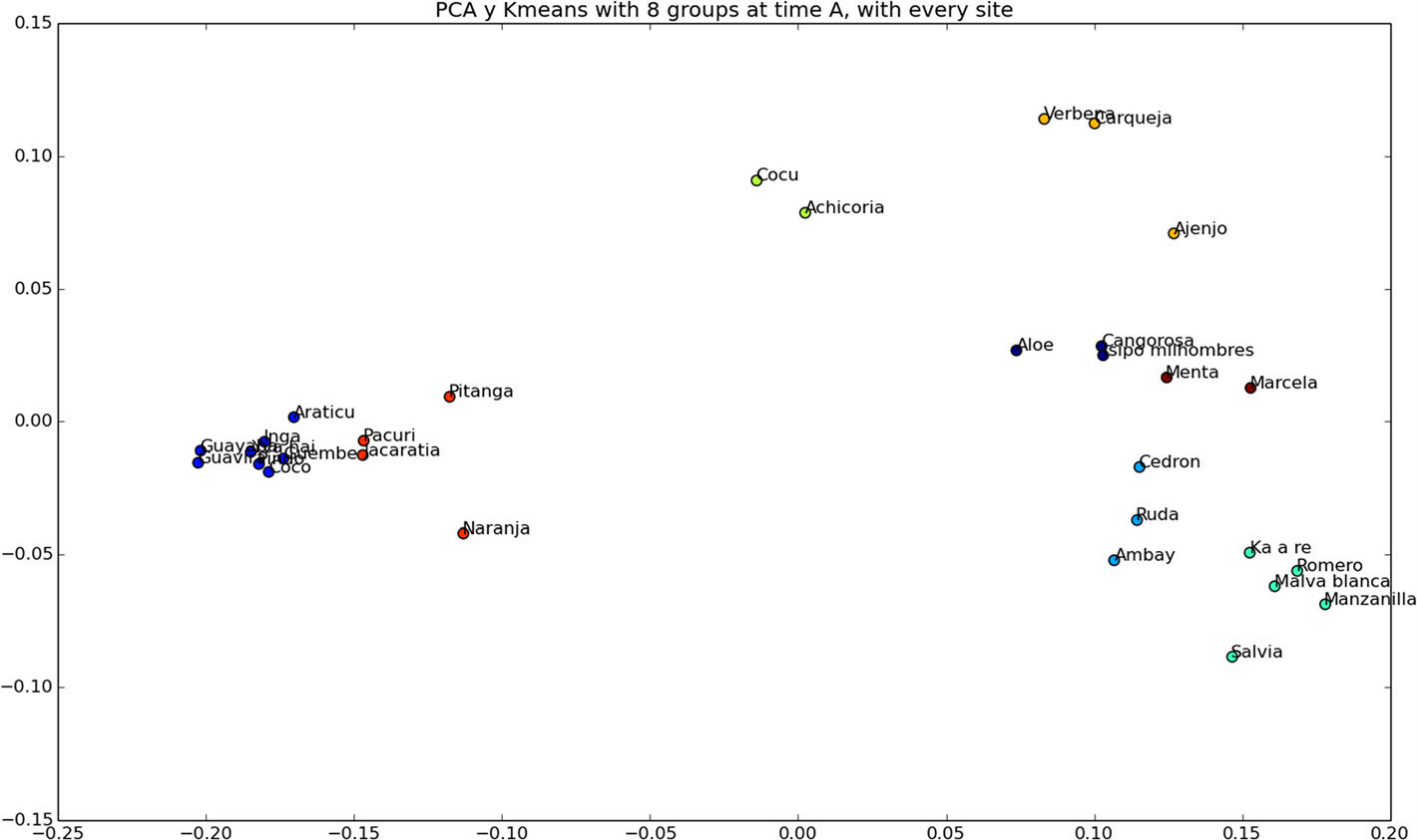
Principal Component Analysis of plant piles sorted by Paraguayan community, Misiones, Argentina

PCA reveals some important patterns in plant categorisation and classification by Paraguayans. The plot (Fig. 6) shows that the plants were categorized according to functional and ecological-morphological attributes. There appears to be an edible (fruit) –medicinal dimension running from left to right across the scaling. Edible plants are clustered on the left side (0.0 divisor vertical line) and medicinal plants are clustered on the right side. At the same time, the left side is dominated by species of fruit trees and two palms (considered as tress by study participants) with one exception – *guembé* (epiphyte), which also bears edible fruits. These plants correspond to wild resources and incipiently domesticated species (with one exception – orange tree – *naranja*). On the other hand, the plants situated on the right side are predominantly herbaceous species, of which most are cultivated or tolerated in home gardens (tree exception on this side are *cangorosa* and *ambay*). Two species situated around this dividing line – *cocú* (fruit tree) and *achicoria* (wild leafy vegetable) are edible species which nonetheless are used more often in the medicinal context. Another dimension, the vertical one, reveals humoral properties of the classified plants, i.e., perceived hot-cold symbolic (not thermal) quality of the plant, which alleviates conditions of the opposite perceived humor [37]. On the top are bitter species such as *achicoria*, *verbena*, *carqueja*, and *ajenjo* along with the cold species such as *cocú* and *verbena*. The hottest plants—*naranja*, *ruda*, *ambay*, *ka a’re*, *romero*, *malva blanca*, *manzanilla*, *salvia—*are situated at the bottom. Therefore, we may surmise that what may appear as purely utilitarian categorisation and classification on the first sight is augmented by ecological concerns (e.g., proximity to the dwelling, state of domestication) and by morphological cues (e.g., plant habit, shape, and coloration). PCA helped us observe groupings and divisions that we were not able to distinguish while analysing clusters, particularly the inter-connection between functionality and morphology. PCA also helped to elucidate that pile sorts were not based on single justification but different dimensions, indexed by different justifications, used side by side.

It is worthy of mention that one informant (who was very knowledgeable about medicinal plants and actively using them, as revealed in the free-list interview) formed only three groupings. Despite her deep knowledge about properties of plants, the informant decided to group plants according to where they grew. Hence one group were species grown naturally in the forest: *cangorosa*, *cocú*, *yva hai*, *jacaratiá*, *pitanga*, *pacurí*, *ambay*, *pindó*, *coco*, *isipó milhombres*, *guavirá*, *ingá*; plants from a home garden: *ka’a re*, *ruda*, *malva blanca*, *ajenjo*, *romero*, *manzanilla*, *salvia*, *aloe*, *cedrón*, *menta*; plants that could grow naturally, but might also be planted in the cultivated field or home garden: *marcela*, *guembé*, *achicoria*, *carqueja*, *verbena*, *guayaba*, *naranja*.

When we compare the general cluster with PCA, it is clear that the edible species are grouped in the following order: *naranja*, *pitanga*, *araticú*, *coco*, *pindó*, *guembé*, *yva hai*, *jacaratiá*, *ingá*, *pacurí*, *guayaba* and *guavirá*. These 12 species comprise a group within the cluster and it is reflected as well in the PCA. One might surmise that both results are congruent and that the study participants have grouped them according to their edibility. Yet in the case of medicinal plants it is not always so clear, because plants can be associated with different illnesses, overlapping sensorial properties, forms of administration, and so forth. It is clear that they are recognized as medicinal but as having different modes of application, so their position can vary accordingly in the cluster or the PCA. This is also reflected clearly in the PCA where edible plants are closely grouped at very short distance, contrary to the medicinal ones that are more broadly scattered in the graph.

### Semantic domains and justification

**Fig. 7:**
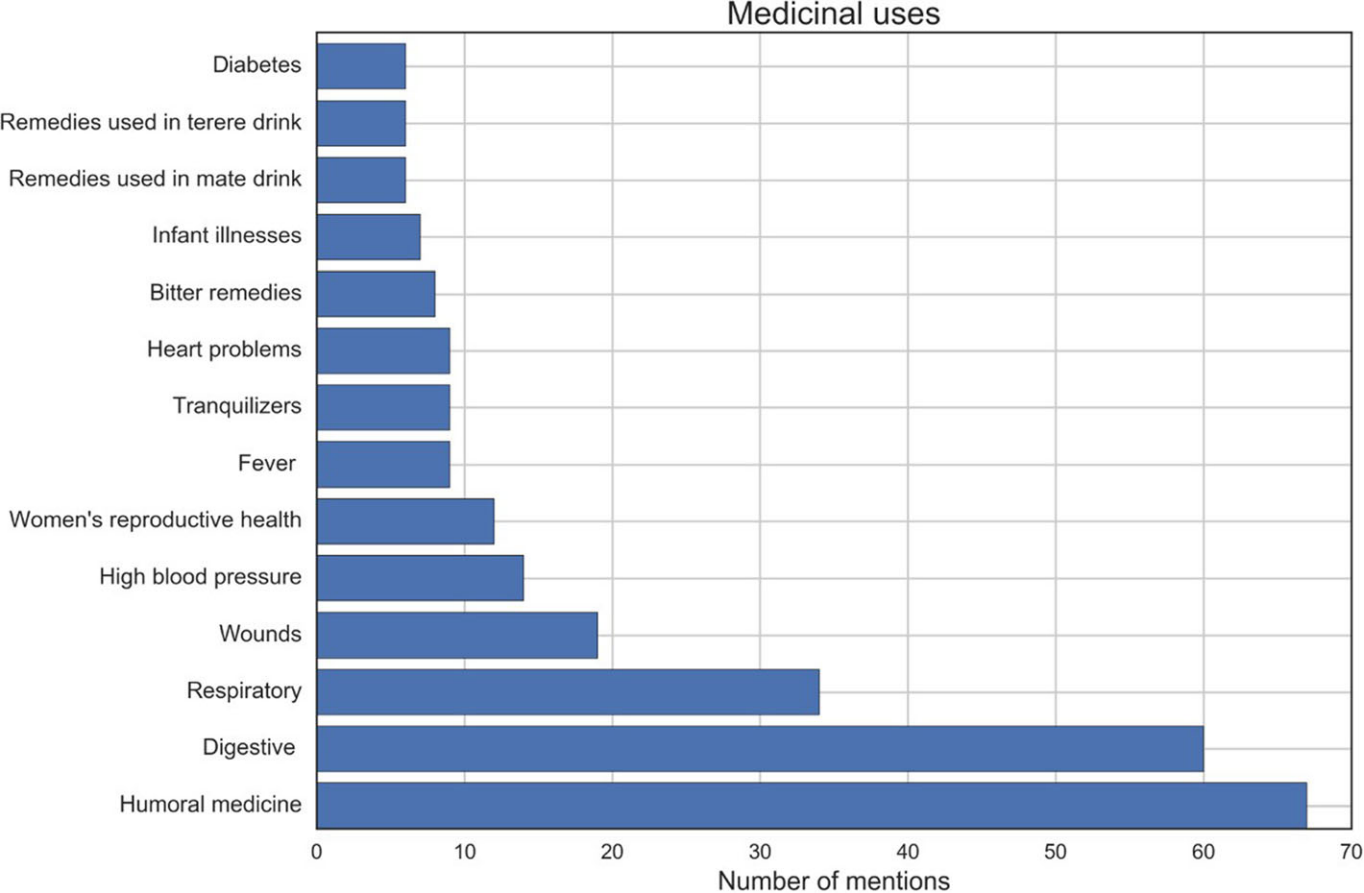
Medicinal uses within plant classification in all study sites, Misiones, Argentina

The overall analysis of all categories found in the plant classification revealed the high salience of medicinal uses (which stays in the line with Clusters and PCA) (Table 2). The medicinal category is not only the most numerous but also the one with the greatest number of uses and properties ascribed to the plants associated therein. The edible domain is much less numerous and less structured than the medicinal category (simply edible, edible fruits, wild edible fruits, salads). Other categories are cultural uses of plants (mainly apotropaic and protection against envy), ecology (grade of domestication) and household uses (ornamental and natural repellents). Morphology with 9 mentions is among the three least mentioned categories. Within morphology domains we included all the explanations for plant grouping provided, based on overall similarity between species—plant form, leaf form, fruit shape, similar taste and the same raping period. Within the medicinal category we followed and adhered to the emic explanations and reasoning provided for plant groupings. Therefore the uses for plants in Fig. 7 are expressions of local terms.

**Table 2:** Categories based on Paraguayans’ justification for pile sorting (all localities)

Etic category – reasons for plant classification	Nr of citations
Medicinal uses and medicinal properties	305
Edible uses	45
Cultural uses	22
Morphology	9
Ecology (grade of domestication)	4
Household uses	2

Humoral concepts, such as hot and cold remedies and blood cleansing were mentioned with the highest frequency from among all justification in the medicinal domain. They were followed by digestive and respiratory health problems. This finding confirms our results from the free-list interviews, where digestive, respiratory problems and humoral concepts (in this order of importance) were mentioned with the greatest frequency. Therefore, to some extent, the task of pile sorting is a fruitful method for data triangulation. However, other important sub-categories in the medicinal domain, based on free listing, include treatment of skin problems, circulatory system disorders, and urinary infections. Instead, plant classification pointed to the importance and validity of humoral concepts in health beliefs. This is perhaps due to the fact that during plant sorting, information is elicited in a different way than during standard ethnobotanical free list tasks, which in our case concentrated on plant uses as responses to health symptoms. During pile sorting, several less tangible or even covert categories acquired importance. It is interesting to see that some relatively new health problems such as high blood pressure and diabetes emerged as relevant during pile sorting. It can be argued that plants with bitter properties could be placed in the morphology domain, but due to the fact that they were mentioned as “remedies”, they were located in medical domain. It is also worthy of mention that a considerable number of groupings were based on forms of preparation and administration. This indicates and highlights the cultural importance of mate and *tereré* as conveyors or vehicles for medicinal plant intake.

Folk nomenclature of species chosen for classification does not reveal any similarities in plant naming. Therefore we could not observe any groupings based simply on similar appellatives.

### Does gender influence plant classification?

**Fig. 8:**
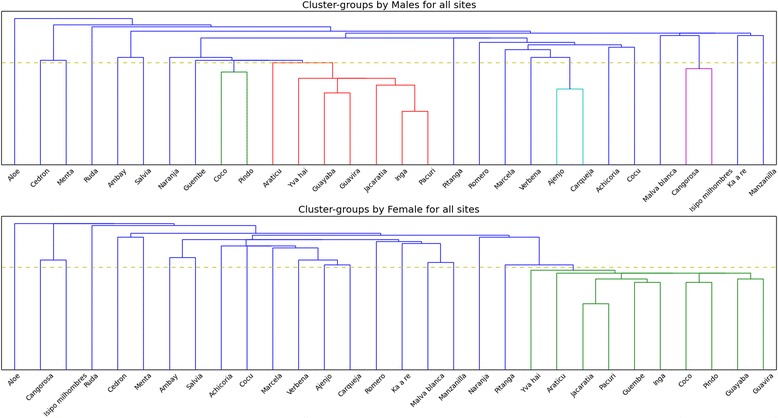
Cluster histogram for Paraguayan men and women in the three study localities, Misiones, Argentina

The clusters produced by men and women are quite similar overall (Fig. 8). Men found tighter connections between edible plants than women did. Men clustered 11 edible plant species together, but *cocú* and *pitanga*, which could be both food and medicine – were clustered with medicine. Women clustered *cocú* with medicinal plants too, but *pitanga* was grouped with edible plants (12 edible species). As for medicinal plants, there are clusters shared by both genders: *cedrón* and *menta* (mate additives, relaxing plants), *ambay* and *salvia* (both used for cough), *achicoria* and *cocú* (cold remedies), *verbena*, *ajenjo* and *carqueja* (bitter plants), *cangorosa* and *isipó milhombres* (blood cleansers). In both clusters aloe stays on its own. The discrepancies are found in different clustering digestive remedies, plants suitable for children and for women, hot remedies. In both clusters we observe the cross-cuttings between functional and perceptual characteristics. Two palms were closely clustered by both genders, also plants from Myrtace family were grouped together: *guayaba*, *guavirá* and *yva hai* (men), *guayaba*, *guavirá* (women). The same is true about typical forest fruit trees. However, it can be concluded that groupings based on morphological similarities are better observed in men’s cluster.

## Discussion

### Goal-derived categories in plant classification

Our results suggest that Paraguayan migrants categorized plants according to cultural utility or goals, specifically uses and properties of plants revealing specific functionality or use-patterns. Apart from forming two basic categories of medicinal and food plants, they also made clusters according to modes of preparation and administration of plant species according to taste (bitter and non-bitter plants). Interestingly, plants were also arranged according to ecological cues (e.g., places where plants grew) and to corresponding life forms. These results are congruent with the conclusions derived by other scholars that folk-classification may reflect a mixture of goal-derived and morphological categories, even in the situation where functionality prevails over other forms of classification [19]. The Paraguayan community in Misiones also proved to have one cohesive system of plant categorization and classification, which stays in line with our hypothesis. The results also suggest that gender does not influence folk classification.

Different motivations and ideas were observed for sorting plants together. On the one side, the justifications for grouping edible plants were basic and clear. Study participants limited them by virtue of their edibility, similar taste or origin from similar environments. Therefore, the sorting task indirectly revealed a limited interest among Paraguayans in exploring these resources, which cannot be said about medicinal plant groupings. Here more individualistic and esoteric knowledge and interests were revealed. However, in both categories of food and medicine we observed multiple justifications in grouping particular plants together. In the medicinal category they were rather based on simultaneous functions (the same species used in different medicinal context) and in food categories they were based more on a combination of concurring functional, ecological and morphological cues. As we could observe, goals serve to activate context-dependent properties [11]. Some species were grouped for sedative properties but at the same time were recognized as excellent additives to mate: mint and lemon grass; or bitter plants may be remedies for digestive problems and hence grouped as a bitter group by one person or as digestive agent by another or one person may give two justifications for grouping.

One explanation here may be that personal experience with plant resources (especially medicinal ones) plays an important role in plant classification. For Paraguayan migrants, category construction is influenced strongly by medicinal needs and a “joy” (intellectual pleasure) of knowing/being familiar with plants and their distinct sensorial and medicinal properties. This reflects clearly an emphasis on cultural necessity and intellectual understandings. The reliance on use-based features can be related to the perceived importance of local medicinal plants. “One of the cultural characteristics (…) most remarkable about Paraguayans of all ages and social conditions – is their daily consumption of medicinal plants. These plants provide health care, nutrients, refreshment and savings on bus fare and expensive visits to doctors” [38]. An interesting outcome of this research is the relevance of humoral medicinal concepts for the study group. Much have been written on the humoral concepts and their resilience in Latin American cultures [37, 39–41]. There has been an ongoing debate about the origin of humoral medicine in Latin America, whether it was an original American concept characteristic to indigenous ethnomedical systems or rather an import from Iberian Peninsula [42, 43]. Our ambitions here are far too modest to contribute to this debate, however our data suggest that humoral medicine, especialy “hot” and “cold” syndromes are strongly present in Paraguayan Mestizos’ illness etiology. Our findings stay in agreement with the conclusion done by Scarpa [41] about *Criollo* (Mestizo) people in the Argentinean Chaco region.

It was already signalled in the Background that the research on ethnobiological classification in the south cone of South America had been under influence of Berlinean ideas [20]. Interestingly, nearly all studies there have concentrated on animal classification. A good review of research performed in this topic may bring a chapter on Ethnociences in *Manual de Ethnozoología* [44]. To our knowledge, there has been just one study performed in Misiones, Argentina so far on ethnobiological classification [45]. Zamudio and Hilgert concentrated on the classification of stingless bee species among farmers of Paraguayan origin in the east-north of Misiones. Their concern, however, was different from ours, as they focused on groupings formed around prototypical taxa, and lingusistic issues, such as representativeness of monomials and binomials in folk nomenclature. Nonetheless, these authors also found functionality explanations, apart from ecological and behavioural, in the process of grouping bees together. Begossi and colleagues [46] who did a comparative study on fish folk taxonomy on the Atlantic Forest Coast and in Amazon, also stated that high-ranking groups called “relatives” are sorted by fishers in both regions principally in terms of similarity of habitat, diet and morphology. However, the mentioned studies concentrated on general classification, hence straight comparison between their research and ours cannot be done.

### Should the scientific concept of morphology be re-articulated in folk epistemology?

Morphology refers to the study of the physical form and external structure of plants. Plant morphology is useful in the formal identification of plants in the taxonomic sense. Morphology is comparative, qualitative and descriptive. In studies of folk systems of knowledge this definition should be reformulated. Humans everywhere presumably recognize and use morphology to recognize plant ethnospecies, Yet our study has shown peoples do not depend upon “morphology” exclusively as a proxy to categorize plants; rather, morphology exists along and within a continuum spanning visual perception, odor (olfactory perception), taste, and touch (i.e. occurrence of more or less hairy leaf surfaces). These organoleptic properties combine to signalize various use-patterns for plants, and as such they transcend other potential signals and factors known to be used in folk classification systems [cf. 18]. The system of classification elicited here articulates with what Medin et al. [19] call a “utilitarian structure imposed on nature rather than a reflection of categories salient in the environment”.

## Conclusions

Our study reveals that familiarity and personal experience with plant resources (especially medicinal ones) play an important if not crucial role in plant classification. The pile sorting task deployed here, based on the most salient species known throughout the region, has advantages over other methods as all study participants are shown to be at least cognizant of all or nearly all of the species included in the task. Accordingly, informants provided meaningful goal-oriented justifications for categorizing plants in this study. However, the categories which emerge through completion of the task are shown to be primarily goal-derived. These results suggest that culturally-constituted modalities take precedence over morphologically-derived approaches to classifying useful flora, which may extend to other ethno-taxa of living things. Furthermore, analyses of pile sort results proved useful for data triangulation and for discovering several less tangible though socially shared dimensions of classification deemed important among members of the study community.

## Abbreviations

PCA: Principal component analysis

## References

[1] Berlin B. Ethnobiological classification: Principles of categorisation of plants and animals in traditional societies. Princeton: Princeton University Press; 1992.

[2] Dwyer PD. Ethnoclassification, ethnoecology and the imagination. Le Journal de la Société Océanistes. 2005;120–121:10–25.

[3] Bernard A (2000). History and theory in anthropology.

[4] Lévi-Strauss C (1962). La pensée sauvage.

[5] Berlin B (1972). Speculations on the growth of ethnobiological nomenclature. Lang Soc.

[6] Berlin B. The Chicken and the egg-head revisited: Further evidence for the intellectualist bases of ethnobiological classification. In: Posey D, Overal W, Clement C, Plotkin M, Elisabetsky E, Novaes da Mota C, Pessoa de Barros J, editors. Ethnobiology: Implications and Applications, Volume 1, Proceedings of the First International Congress of Ethnobiology, Belem, 1998. Belem: Museu Paraense Emflio Geoldi; 1990. p. 19–33.

[7] Brown C. Language and Living Things: Uniformities in Folk Classification and Naming. Brunswick: Rutgers University Press; 1984.

[8] Brown C (1986). The growth of ethnobiology nomenclature. Curr Anthropol.

[9] Hunn E (1982). The utilitarian factor in folk biological classification. Am Anthropol.

[10] Randall RA, Hunn E (1984). Do life forms evolve or do uses for life: Some doubts about Brown’s universal hypothesis. Am Ethnol.

[11] Balée W (1999). Footprints of the Forest. Ka'apor Ethnobotany—the Historical Ecology of Plant Utilization by an Amazonian People.

[12] Ingold T, Näripea E, Sarapik V, Tomberg J (2006). Up, across and along. In: Place and location. Studies in environmental aesthetics and semiotics. Place and location. Studies in environmental aesthetics and semiotics.

[13] Rival L, Alexiades MN (2009). Towards an understanding of the Huaorani ways of knowing and naming plants. Mobility and migration in indigenous Amazonia.

[14] Boster JS, Dougherty J (1985). “Requiem for the omniscient informant”. There’s life in the old girl yet. Directions in cognitive anthropology.

[15] Boster JS, Johnson JC (1989). A comparison of experts and novice judgments in similarity among fish. Am Anthropol.

[16] Nolan JM (2001). Pursing the fruits of knowledge: Cognitive ethnobotany in Missouri’s Little Dixie. J Ethnobiol.

[17] Nolan JM. Wild plant classification in little Dixie: Variation in a regional culture. J Ecol Anthropol. 2002;6(1):69–81.

[18] Nolan JM. Wild harvest in the heartland: Ethnobotany in Missouri’s Little Dixie. Lanham: Rowman and Littlefield; 2007.

[19] Medin DL, Lynch EB, Coley JD (1997). Categorization and reasoning among tree experts: do all roads lead to Rome?. Cogn Psychol.

[20] Soares WFJ, Santos Gonçalves PH, Paiva de Lucena RF, Albuquerque UP, Albuquerque UP, RRN A (2015). Alternatives ways of folk classification. Introduction to Ethnobiology.

[21] Mourão JS, Araujo HFP, Almeida FS. Ethnotaxonomy of mastofauna as practiced by hunters of the municipality of Paulista, state of Paraíba-Brazil. J Ethnobiol Ethnomed. 2006;2:19. 10.1186/1746-4269-2-19 PMC1473038 16603080

[22] Souza SP, Begossi A. Whales, dolphins or fishes? The ethnotaxonomy of cetaceans in São Sebastião, Brazil. J Ethnobiol Ethnomed. 2007;3:9. 10.1186/1746-4269-3-9 PMC1804260 17311681

[23] Barsalou LW, Ross BH. The role of automatic and strategic processing in sensitivity to superordinate and property frequency. J Exp Psychol Learn Mem Cogn. 1986;12:116–34.

[24] Clément D (1995). Why is taxonomy utilitarian?. J Ethnobiol.

[25] Quinlan M (2005). Considerations for collecting freelists in the field: examples from ethnobotany. Field Methods.

[26] INDEC. Censo Nacional de Población, Hogares y Viviendas 2010. Argentina: Instituto Geográfico Nacional (IGN). http://www.sig.indec.gov.ar/censo2010/. Accessed 10 Feb 2014.

[27] Torres VE (2014). Paraguayos en Argentina: Propensión a emigrar y características sociodemográficas (2001-2010). Folia Histórica Nordeste.

[28] La inmigración limítrofe. In: Torrado S, editor. Población y bienestar en la Argentina del primero al segundo Centenario. Una historia social del siglo XX, vol. I. Buenos Aires: Ensayo Edhasa; 2007. p. 571–99.

[29] Galindo-Leal C, Câmara IG, Galindo-Leal C, Câmara IG (2003). Atlantic Forest Hotspot Status: An Overview. The Atlantic Forest of South America: biodiversity status, threats, and outlook.

[30] Da Ponte E, Roch M, Leinenkugel P, Dech S, Kuenzer C (2017). Paraguay’s Atlantic Forest cover loss – Satellite-based change detection and fragmentation analysis between 2003 and 2013. Appl Geogr.

[31] Weller SC, Romney AK. Systematic Data Collection. Thousand Oaks: Sage Publications; 1988.

[32] Martin G (1995). Ethnobotany: a method manual.

[33] Jones E, Oliphant E, Peterson P, et al. SciPy: Open Source Scientific Tools for Python, 2001-, http://www.scipy.org/. Accessed 21 July 2016.

[34] Tibshirani R, Walther G, Hastie T (2000). Estimating the number of clusters in a data set via the gap statistic. J R Stat Soc Ser B.

[35] Krippendorf K (2013). Content analysis: an introduction to its methodology.

[36] Silverman D (1993). Interpreting qualitative data: strategies for analysing talk, text, and interaction.

[37] Messer E (1987). The hot and cold in Mesoamerican indigenous and Hispanicized thought. Soc Sci Med.

[38] Moreno NB (2007). The role of the medicinal plants in rural Paraguayan livelihoods reasons for extensive medicinal plant use in Paraguay. Suplemento Antropológico, Revsta de Centro de Estudios Antropológicos Asunción, Paraguay.

[39] Foster GM (1953). Relationships between Spanish and Spanish-American folk medicine. J Am Folk.

[40] Quinlan MB, Quinlan RJ, Pieroni A, Price LL (2006). Balancing the system: humoral medicine and food in Commonwealth of Dominica. Eating and healing. Traditional food as medicine.

[41] Scarpa GF (2004). El síndrome cálido-fresco en la medicina popular criolla del Chaco argentino. Dialectología y Tradiciones Populares.

[42] Foster GM, Wetherington RK (1978). Hippocrates’ Latin American Legacy: ‘Hot’ and ‘cold’ in contemporary folk medicine. Colloquia in Anthropology 2.

[43] López AA. Cuerpo Humano e ideología, vol 1. Mexico: UNAM; 1980.

[44] Costa Neto EM, Santos Fita D, Costa Neto EM, Santos Fita D, Vargas Clavijo M (2009). Etnocienciass: la busqueda por categorías de realidad. Manual de etnozoología. Una guía teórico-práctica para investigar la interconexión del ser humano con los animales.

[45] Zamudio F, Hilgert N (2015). Multi-dimensionality and variability in folk classification of stingless bees (Apidae: Meliponini). J Ethnobiol Ethnomed.

[46] Begossi A, Clauzet M, Figueiredo JL, Garuana L, Lima RV, Lopes PF, Ramires MF, Silva AE (2008). RAM Silvano. Are biological species and higher-ranking categories real? fish folk taxonomy on Brazil's Atlantic forest coast and in the Amazon. Curr Anthropol.

